# Knowledge and attitude toward depression among healthcare professionals working in Ilu Aba Bor zone, Oromia regional state, Ethiopia, 2021: a cross sectional study

**DOI:** 10.3389/fpsyt.2023.1059698

**Published:** 2023-11-29

**Authors:** Yadeta Alemayehu, Million Girma, Lemi Bacha, Kefale Boka, Hunde Tarafa

**Affiliations:** Department of Psychiatry, College of Health Sciences, Mattu University, Mattu, Ethiopia

**Keywords:** knowledge, attitude, depression, health care professionals, Ethiopia

## Abstract

**Background:**

Depression is the major mental disorder that frequently co-occurs with other physical illnesses, although its detection at primary healthcare is limited. Thus, the purpose of this study is to evaluate health professionals’ knowledge and attitude toward depression and its related factors.

**Objective:**

To assess knowledge and attitude of healthcare professionals toward people with depression at different health facilities of Ilu Aba Bor zone, Ethiopia, 2021.

**Methods:**

A cross-sectional study was carried out involving 404 primary healthcare professionals using a systematic random sampling technique in February 2021. Attitude was assessed using depression attitude questionnaires. Epi-data version 3.1 and SPSS version 26 was used for data entry and analysis. The *p*-values lower than 0.05 were deemed statistically significant.

**Result:**

In the current study, 30.4% (95% CI; 25.86, 34.94) of the respondents have inadequate knowledge and 29.9% (95% CI; 25.4, 36.8) have negative attitude toward depression. A vast majority (77.7%) of healthcare professionals have never received any kind of training on mental health. Accordingly, contact with the person with mental illness and mental health training were significantly associated with inadequate knowledge. In addition, perceived cause of depression and mental health training were significantly associated with negative attitude.

**Conclusion:**

According to this study, the magnitude of inadequate knowledge and negative attitude in the diagnosis and treatment of depression is comparably high. Therefore, primary healthcare settings should prioritize raising awareness, promoting positive attitudes, and improving detection and treatment of depression cases.

## Introduction

Depression is a common mental illness marked by low mood, a lack of interest or enjoyment, a sense of unworthiness or unwarranted guilt, interrupted sleep, disordered appetite, feeling fatigued, poor concentration, and suicidal ideation (1). Negative life events, including the end of a meaningful person–object relationship or a decline in health, frequently result in depression. These issues might become severe over time or become recurring, which can seriously affect a person’s capacity to handle daily responsibilities (2).

According to a 2017 WHO report, depression affects more than 300 million people worldwide of all ages (3). By the year 2030, depression, which is currently ranking third, is expected to surpass heart disease as the second leading cause of global illness burden in the world (4). Depressed individuals have twenty times greater mortality rate due to suicide than the general population (5, 6). In Ethiopia, depression is among the top ten most burdensome ailments, surpassing HIV/AIDS and accounting for around 6.5% of the total disease burden (7, 8). Common mental disorder (consisting depression, anxiety, and somatic disorders) is most prevalent in the research region, Ilu Aba Bor zone, with a prevalence of 27.2% (9).

The World Health Organization (WHO) describes how to incorporate mental health treatments into the context of basic care. Education is essential for enhancing the commencement of supportive therapies, increasing referrals to more specialist healthcare professionals, and improving the detection of serious mental disorders in basic healthcare. This service heavily relies on the ability of medical practitioners to recognize and treat serious mental illnesses such as depression (10, 11).

There have been allegations of health professionals stigmatizing people with severe mental disorders (SMD), through delivering words of discouragement, making negative statements, rejecting behavior, and having negative attitudes (12, 13). Given this stigmatiztion, it has been suggested that the mental health literacy of healthcare professionals on depression is a significant driver of the quality and inclusiveness of care for those with depressive disorders (14).

Lack of understanding of depressive illnesses, including information about their symptoms and treatments, has an effect on the provision of psychiatric services. Studies demonstrate that the levels of stigma decrease as knowledge increases. In addition, attitudes might range from positivity and tolerance to negativity and terror. A supportive and receptive attitude develops when a good attitude is displayed. Conversely, negative views lead to avoidance, social marginalization, and discrimination (15).

More than 45% of the world’s population reside in a nation with less than one psychiatrist for every 100,000 citizens, according to the WHO Mental Health Atlas 2014. However, if treatments for those suffering from mental, neurological, and substance use disorders were only provided by professionals, millions would be unable to access the necessary help (16). To create community mental health services, WHO has suggested integrating mental health within the current primary healthcare system and utilizing local resources (17).

Sub-Saharan Africa has a dearth of personnel to support its healthcare systems. Strategic primary care structures have developed to provide healthcare to the populace, but there is a considerable concern regarding their effectiveness and ability to function in situations with limited resources. The dearth of specialists reinforces the fact that primary mental healthcare needs to be strengthened in sub-Saharan Africa (18). Additionally, less than 2% of the overall health budget is allocated to mental health, and in low-and middle-income countries (LMICs), more than 75% of those with mental disorders do not receive any type of modern therapy (19). For the community’s overall health, the World Health Organization advises that primary healthcare centers should include mental health services (20). However, barriers to integrating mental healthcare into primary healthcare (PHC) units have been identified, including attitudes, knowledge, and abilities related to delivering mental health services (21).

The newly emerged COVID-19 coronavirus has caused traumatic stress due to isolation or physical distancing, job loss, and family members falling ill of COVID-19. Due to the impact of the pandemic, the prevalence of common mental disorder is highly increasing. For this reason, it is important to determine the knowledge and attitude level of health professionals to identify, diagnose, and manage illnesses early (22, 23).

Depressive disorder often co-occurs with other medical illnesses. Approximately 24%–81% of patients visiting health institutions for medical problems have comorbid depression (24–27). A study in Ethiopia also revealed the prevalence of common mental disorder among outpatient medical patients is 39.2% (28), and the prevalence of depression among medical outpatients is 24% (27). Even though mental disorders co-exist with other medical problems, negative attitude and stigma related with mental illness limit patients from taking comprehensive and holistic care at healthcare institutions (29, 30).

Health professionals’ knowledge of mental illness has been a crucial factor for quality and inclusive care for those with mental illness due to stigmatizing attitudes of healthcare personnel toward patients with SMD (13). In sub-Saharan African contexts, the feasibility of integrating mental health in to PHC is limited due to inadequate mental health knowledge, unfavourable expectations of PHC staff and other health system factors (31).

According to Ethiopia’s national mental health policy, Health Extension Workers (HEWs) should engage in promotion and prevention activities in their neighborhoods to raise awareness of mental health issues, reduce stigma, and encourage the use of mental health services. Employees at the healthcare centers are required to recognize, screen, diagnose, treat, and keep an eye on people who have mental illnesses. This includes writing prescriptions for psychotropic drugs, giving brief psycho-educational and psychosocial interventions, and making referrals as needed. However, there is little attention paid to mental diseases, and the majority of mental health services are still provided solely by psychiatry specialists in hospitals (32).

The majority of studies conducted around the world demonstrate the seriousness of the problem. A study conducted in Lebanon revealed the prevalence rates of unfavorable knowledge, low attitude, and behavior to be 67%, 67.8%, and 73.1%, respectively (33). A study in Kenya revealed the prevalence rates of unfavorable knowledge and low attitude to be 62.25% and 63.22%, respectively (12).

A study conducted in Ethiopia in a primary healthcare setting showed that 50%of healthcare providers had unfavorable knowledge, while 44.2% had a negative attitude (34). Another study in Ethiopia revealed that approximately 48.2% of healthcare providers have low attitude toward patients with mental illness (35). Different studies among primary healthcare providers in Ethiopia showed a prevalence of unfavorable knowledge, low attitude, and behavior. In Amhara region, the prevalence rates were found to be 28.1%, 34.5%, and 40%, respectively (36). In Addis Ababa city, the rates were even higher, with 66%, 93.4%, and 24.8% for unfavorable knowledge, low attitude, and poor behavior, respectively (37).

Even though the Minister of Health of Ethiopia intended for mental health services to be evaluated and addressed in the primary healthcare settings, studies on mental health literacy toward mental illnesses, including depression in Ethiopia, revealed the existence of gaps in knowledge and an unfavorable attitude among healthcare professionals. This study provided information for policy briefers, evaluators of mental health services, and healthcare management concerning the level of clinician knowledge and attitude about depression.

To the best of the researchers’ knowledge, there is a lack of information that explains the level of knowledge and attitude of mental health service providers in Ethiopia and the study location. Despite studies in various fields indicating that health providers have unfavorable knowledge and a negative attitude toward depressive disorders in primary healthcare, there are no specific data available for this situation. Therefore, the purpose of this study is to assess the level of knowledge and attitude of healthcare professionals in the Ilu Aba Bor zone of Oromia, Ethiopia. Furthermore, the study examined different factors related to the level of inadequate knowledge and unfavorable attitudes of health professionals.

## Materials and methods

### Study area

The investigation was carried out in the southwest Ethiopian province of Oromias’ Ilu Aba Bor zone. The zone’s capital, Mettu town, lies 600 km away from Addis Ababa in the south-west of Ethiopia. It covers the western part of the region and lies between 340 52′12″ E to 410 34′ 55″ E longitude and 70 27′ 40″ N to 90 02′ 10″ N latitude. The zone is bordered by Gambela Region in the west, Bunno Bedelle Zone in the southeast, West Wollega in the north, East Wollega in the northeast, and Southern Nations, Nationalities and Peoples’ Region (SNNPS) of the country. The zones had been divided into three major climatic conditions: temperate rainy, rainy, and dry arid. The rainy season occurs in both zones during the summer months of June, July and August. The maximum mean annual rainfall is over 2,400 mm, while the minimum is nearly 100 mm. The temperature is moderate despite its high altitude in a tropical climate modified by its altitudinal location in both zones. The highest mean annual temperature ranges from 26°C to 10.6°C in the highland areas of the zones. Ilu Aba bora zone has 1 town administration and 14 rural districts with estimated total population of 1,606,502.

In the Illu Aba Bor zone, there are 286 health posts, 40 health centers, 1 district hospital, and 1 referral hospital. A total of 1,318 health professionals work at these various facilities. Twenty health centers and one district hospital were selected for the study’s goals.

### Study design and period

A facility-based cross-sectional study design was conducted in February 2021.

### Population

Health professionals working at selected primary healthcare facilities of Ilu Aba Bor zone were the study population in this study. All health professionals working at selected primary healthcare facilities of Ilu Aba Bor zone, who were available during the study period, were included in this study. The study participants were health professionals who directly provided clinical service for patients, including public health officers, nurses, midwives, pharmacists, general practitioners, and various medical specialties. The median monthly salary of the participants was ETB 6193 or USD$ 115 (i.e., reported in the Result section).

### Sample size and sampling technique

The sample size was calculated using the single population proportion formula;


$$n=\frac{Z_{\frac{\alpha }{2}}^{2}p\left(1-P\right)}{d^{2}}\text{,}$$

Where,

- *P* is the population proportion of knowledge and attitude among primary healthcare professionals. The study revealed that 50% and 55.8% have inadequate knowledge and unfavorable attitude, respectively (34). Sample size was calculated by considering:
- Margin of error = 0.05 unit
- *Zα*/2 = *Z* value at (α = 0.05) = 1.96 corresponding to 95% confidence level
- 5% of non-response rate

The final calculated sample size was 404 and 398 for knowledge and attitude levels, respectively. Finally, the maximum sample of 404 was taken as a maximum sample size so that it increases the precession and power of the study.

A multistage sampling technique was used to select representative samples of health service providers. There were approximately 1,318 health professionals working at two hospitals, 40 health centers, and 286 health posts in Ilu Aba Bor zone of Oromia region. Twenty health centers and one district hospital from total health facilities were randomly selected and included in the study. A list of health professionals were taken from health facilities, and the sample was proportionally allocated for the health facilities. Finally, data were collected from 404 health professionals.

### Dependent variables

- Knowledge (adequate/inadequate)
- Attitude (Favorable/unfavorable)

### Independent variables

- **Sociodemographic factors:** Sex, age, marital status, educational status, professional qualification, working department, work experience, and income
- **Psychosocial and behavioral factors:** Substance use, contact with the person with mental illness, history of mental illness, family history of mental illness, availability of psychotropic medications, and perceived cause of depression.
- **MH-GAP training**

### Operational definition

- **Adequate knowledge**: If the respondent answers correctly with a mean score of 15 or greater using NIMHANS questionnaires (38).
- **Favorable attitude**: If the respondent answers correctly with a mean score of 20 or greater on depression attitude questionnaires (39).
- **History of mental illness**: If the participant currently has or had any follow-up appointment in any psychiatric clinic.
- **Family history of mental illness:** If there are any follow-up appointments with any member of the family in any psychiatric clinic.
- **mh-GAP training**: WHO designed training for scaling up services for priority mental, neurological, and substance use disorder (40).
- **Current substance use:** Those who have used at least one substance (alcohol, Khat, cigarette, and others) without a medical reason within the last 3 months (41).
- **Contact with the person with mental illness:** It was assessed by asking professionals whether they had a contact with someone with a mental health problem in the past 12 months (42).

### Data collection instruments

The data were collected from healthcare providers by using standard self-administered questionnaires. Self-administered structured questionnaires, a Likert scale, and a semi-structured questionnaire were also used. Questions that explored the participants’ demographic information, thus giving the researcher an insight into biographical information of the respondents, was designed by the investigator. This section enquired about participants’ age, gender, religion, marital status, qualification, mental health training, work experience, past experience working at a psychiatric unit, knowing someone other than the patient with psychiatric conditions, and personal history of mental illness. Additionally, substance use was assessed as an independent factor.

Health workers’ knowledge of depression was assessed using a modified 15-item tool developed by the Department of Psychiatry at the National Institute of Mental Health and Neurosciences (NIMHANS) in Bangalore (38).

Attitude was assessed by 20-item depression attitude questionnaires, with a response of (agree, disagree, and do not know). A single overall score was calculated by summing each individual item, where a high overall score indicates a more negative attitude, with a possible score range of 22–110 (39). For this study, we categorized using the mean scores after checking the normality of the distribution.

### Data collection methods

The study contains quantitative variables which are self-administered and filled by the participating health professionals. Sociodemographic variables were collected using a semi-structured questionnaire, and other variables such as knowledge and attitude were assessed by standard tools. Psychiatry professionals were recruited for data collection and supervision of the data collection activity. A two-day training was given for data collectors and supervisors by the principal investigators on the methods of data collection and the detail of questionnaires.

### Data quality assurance

To ensure uniformity, every questionnaire was translated into Afaan Oromo and then back into English. Pre-testing of the questionnaire was conducted on 5% of the sample’s healthcare professionals in the Bunno Bedelle Zone who were not involved in this study. This pre-test was created to verify the questionnaire’s clarity and applicability, highlight any challenges encountered during application, and estimate the amount of time needed to complete the questionnaire. In accordance with the findings, the necessary adjustment was made. Participants in the study received orientation on how to complete the surveys. During the data collection process, supervisors and primary investigators closely monitored and followed up.

### Data processing, analysis, and interpretation

Coded and checked data were entered into the computer using EPI Data version 3.1 and imported to Statistical Package for the Social Sciences (SPSS) window software version 26 for analysis. To provide a visual representation of the data, descriptive statistics including frequency, percentage, mean, and median were calculated and presented using tables and charts. After checking the normality of the data, the knowledge and attitude levels were manipulated using a mean score. Bivariable and multivariable binary logistic analyses were conducted, and *p*-values lower than 0.05 were considered statistically significant, and the strength of the association was presented by adjusted odds ratio with 95% CI.

### Ethical consideration

This study was conducted in accordance with the Declaration of Helsinki. An official letter or ethical permission was acquired from the College of Health Sciences of Mattu University. Participants provided their informed consent to participation in this study. During the study, confidentiality of the data and response privacy were maintained.

## Results

### Sociodemographic characteristics of respondents

A total of 391 people took part in the study, yielding a response rate of 96.8%. The respondents’ average age (SD) was 30 (3.9). A total of 259 participants (59.1%) were men, and majority of respondents, that is, 285 (71.9%) of them, were married, making up more than half of the participants, and 226 (57.8%) of them identified as protestants. Approximately 164 (41.9%) of the respondents were nurses in their profession and regarding education level of the respondents, and 234 (59.8%) participants graduated with a bachelor’s degree. The median monthly salary of the participants was ETB 6,193 or USD$ 115 (Table 1).

**Table 1:** Sociodemographic characteristics of healthcare providers working at health facilities of Ilu Aba Bor zone, Oromia, Ethiopia, 2021 (*n* = 391).

Variables	Frequency (*N* = 391)	%
Sex		
Male	231	59.1
Female	160	40.9
Age		
18–24	19	4.9
25–34	328	83.9
≥45	44	11.3
Religion		
Orthodox	100	25.6
Muslim	65	16.6
Protestant	226	57.8
Marital status		
Married	285	27.1
Single	106	72.9
Professional qualification		
Nurse	164	41.9
Midwifery	71	18.2
Public health	90	23
Others	66	16.9
Level of education		
Diploma	159	40.2
Bachelor degree and above	234	59.8
Experience in years		
<5 years	125	32
5 years and above	266	68
Monthly income		
≥3792EBr (96$)	277	70.80%
<3792EBr (96$)	114	29.20%

Others* = General practitioners, pharmacy professionals. 1$ = 39.5EBr.

## Description of information regarding healthcare providers

### Prior mental health training

**Figure 1:**
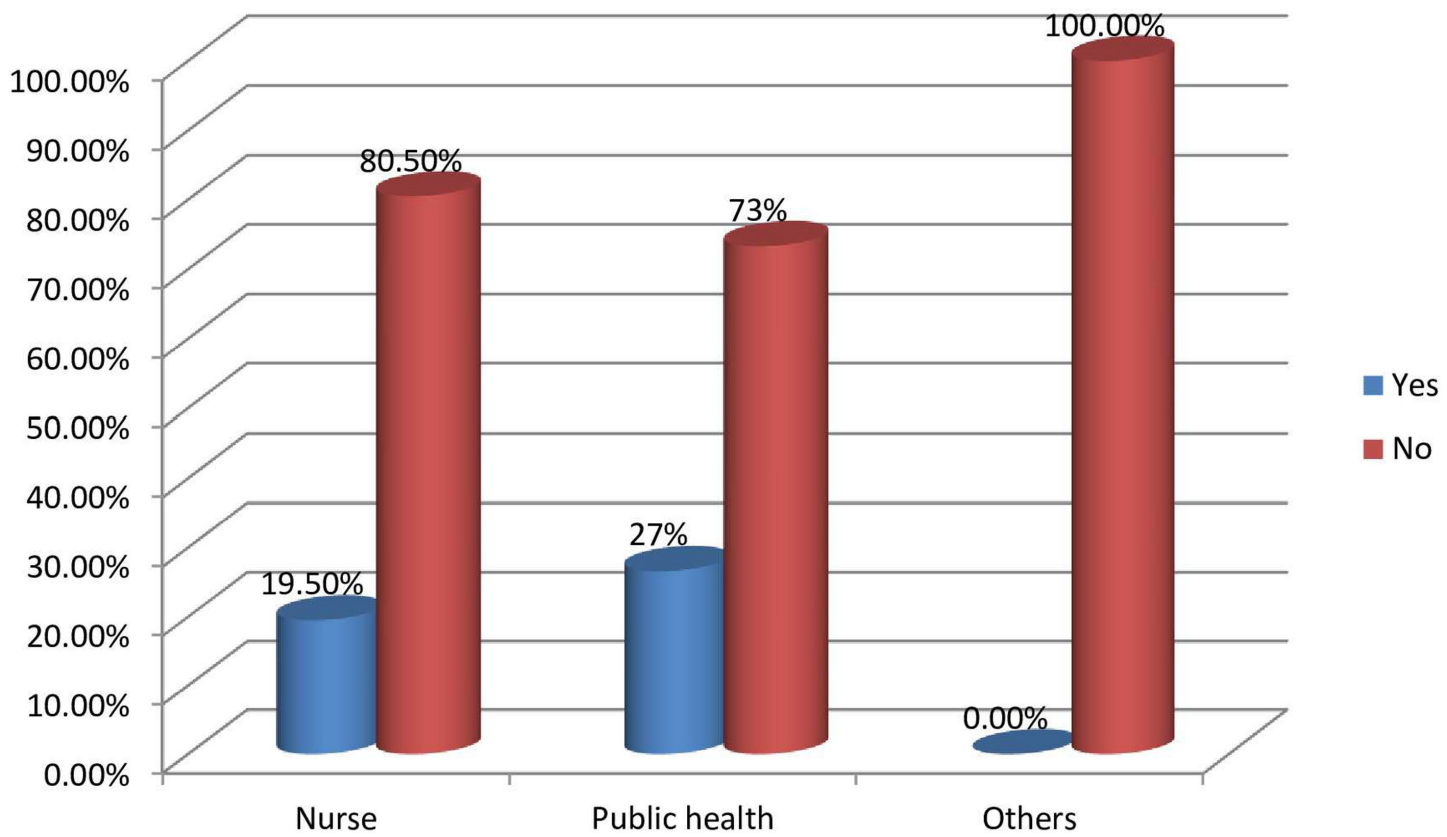
A diagrammatic presentation of mhGAP training status of health professionals in their respective departments.

A vast majority (77.7%) of HCPs had never received any kind of training on mental health. Only a few (10.2%) gained theoretical knowledge and 12% were exposed to clinical practice in previous program. Only a few (14.3%) of the respondents reported taking mhGAP training previously. Among those received mhGAP only nurses (19.5%) and public health professionals (26.6%) were involved (see Figure 1).

### Family history of mental illness

Approximately 12.5% of HCPs reported a family history of mental illness. Among them, more than half reported siblings with mental illness and few reported parental history of mental health problems.

### Availability of antidepressant drugs in the facility

Only 21% of HCPs reported that antidepressant medications are available in their health facility; and respondents mentioned the availability of only amitriptyline at their health facilities (Table 2).

**Table 2:** Other information of healthcare providers working at primary healthcare facilities of Illu Aba Bor zone.

No				*N*	%
01	What kind of training do you received on mental health?	Never trained		304	77.7
		Theory only		40	10.2
		Both theory and clinical practice		47	12
02	Do you have family history of mental illness?	Yes		49	12.5
		If yes, specify	Father or mother	7	14.3
			Siblings	26	53.1
			Others	16	32.7
		No		342	87.5
03	Is antidepressant medication available in your health facility?	Yes (i.e., amitriptyline)		82	21
		No		309	79
04	Do you have previous history of medically diagnosed depressive disorder?	Yes		12	3.1
		No		379	96.9
05	Do you know someone with psychiatric conditions?	Yes		261	66.8
		No		130	33.2
06	Do you have any experience working in psychiatric unit in the past?	Yes		65	16.6
		No		326	83.4
07	Have you ever taken mhGAP training?	Yes		56	14.3
		No		335	85.7

Others: uncle, aunt, son, or daughter.

### Knowledge of the healthcare providers about depression

**Figure 2:**
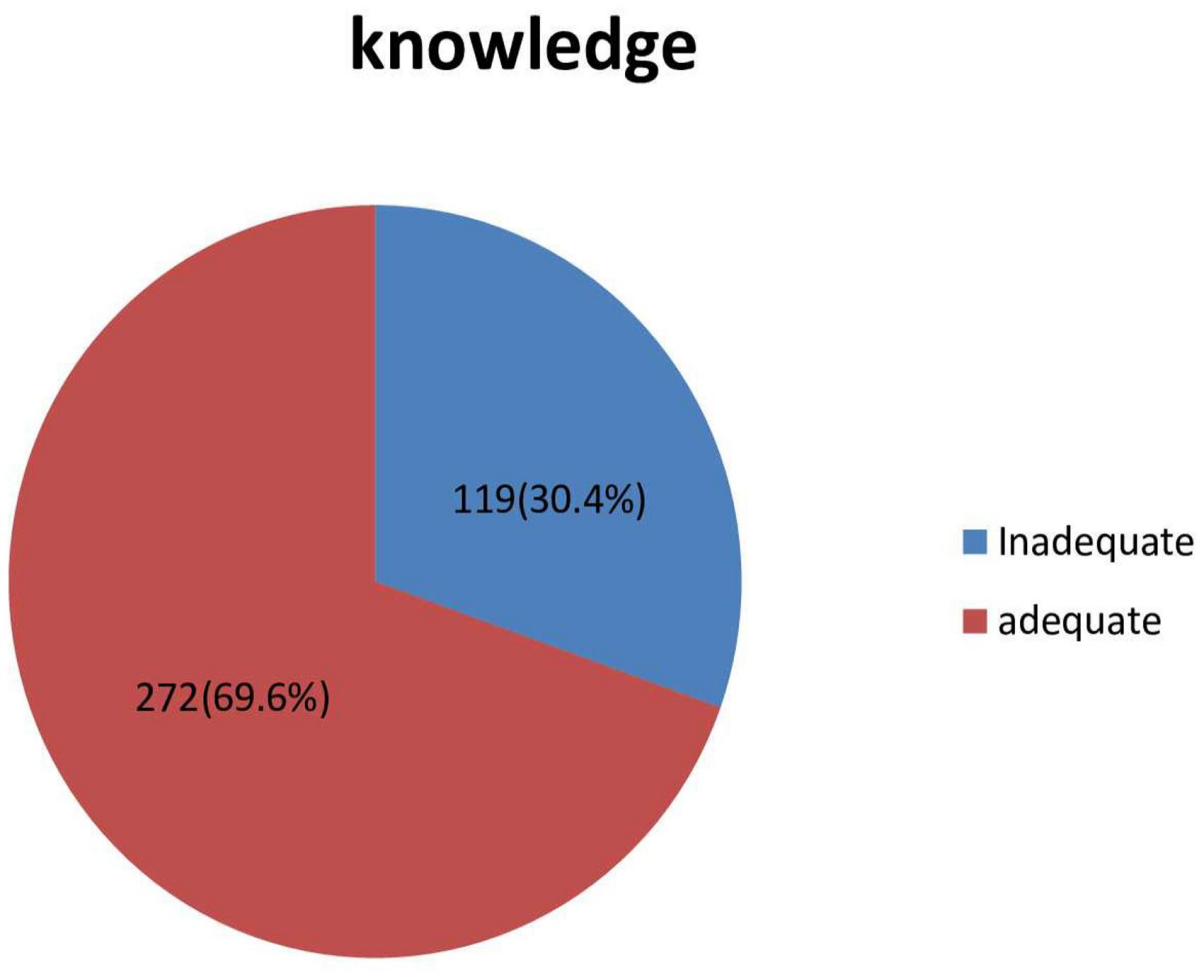
A diagrammatic presentation of magnitude of inadequate knowledge.

Using a mean score as a cutoff point, 119 (30.4%) of the respondents have inadequate knowledge (Figure 2).

The majority of respondents (84.1%) viewed depression as a health issue. Among them, 69.1% respondents rejected the idea that witchcraft, charms, or evil spirits are to blame for depression. Approximately 91.8% of medical professionals said that depression can result in suicide or suicide attempts.

The majority (91.8%) agreed that psychotherapy and pharmaceutical techniques can be used to treat depression. Nearly one-third (31.7%) of the respondents conclude that depression is best managed by traditional doctors/healers. Approximately 50% of the respondents accepted that methotrexate is an anti-depressant drug, while 65% accepted that carbamazepine is one. Approximately 40.4% of the healthcare providers either disagree or do not know that fluoxetine is an anti-depressant drug. Only 22.5% of the respondents knew that a tool was used for classifying depressed patients; however, none of them mentioned that any tool was used for classifying depression (Table 3).

**Table 3:** Description of knowledge of healthcare workers working at healthcare facilities of Ilu Aba Bor zone.

No	Variables	Agree *n* (%)	Disagree *n* (%)	Do not know *n* (%)
01	Have you ever heard about depression?	359 (91.8)	20 (5.1)	12 (3.1)
02	Do you consider depression as a health problem?	329 (84.1)	62 (15.9)	–
03	Depression affects people of a particular age group	83 (21.2)	300 (76.7)	8 (2)
04	Depression is caused by witchcraft, charms or evil spirits	101 (25.8)	270 (69.1)	20 (5.1)
05	Patients with depression can break down at any time	207 (52.9)	178 (45.5)	6 (1.5)
06	Patients with depression are dangerous to themselves and others	314 (80.3)	77 (19.7)	–
07	Depression can lead to suicide and suicide attempts	359 (91.8)	32 (8.2)	–
08	Depression can be treated with pharmacological methods and psychotherapy	359 (91.8)	24 (6.1)	8 (2)
09	Depression is best managed by traditional doctors/healers	267 (68.3)	95 (24.3)	29 (7.4)
10	Depression responds better to traditional remedies than orthodox treatment most of the time	103 (26.3)	247 (63.2)	41 (10.5)
11	Amitriptyline is an anti-depressant drug	341 (87.2)	15 (3.8)	35 (9.0)
12	Methotrexate is an anti-depressant drug	199 (50.9)	114 (29.2)	78 (19.9)
13	Fluoxetine is an anti-depressant drug	233 (59.6)	66 (16.9)	92 (23.5)
14	Carbamazepine is an anti-depressant drug	249 (63.7)	100 (25.6)	42 (10.7)
15	Do you know of a tool used to classify depressed patients? If response is yes, please specify the tool	88 (22.5)	214 (54.7)	89 (22.8)

### Attitude of healthcare providers towards depression

**Figure 3:**
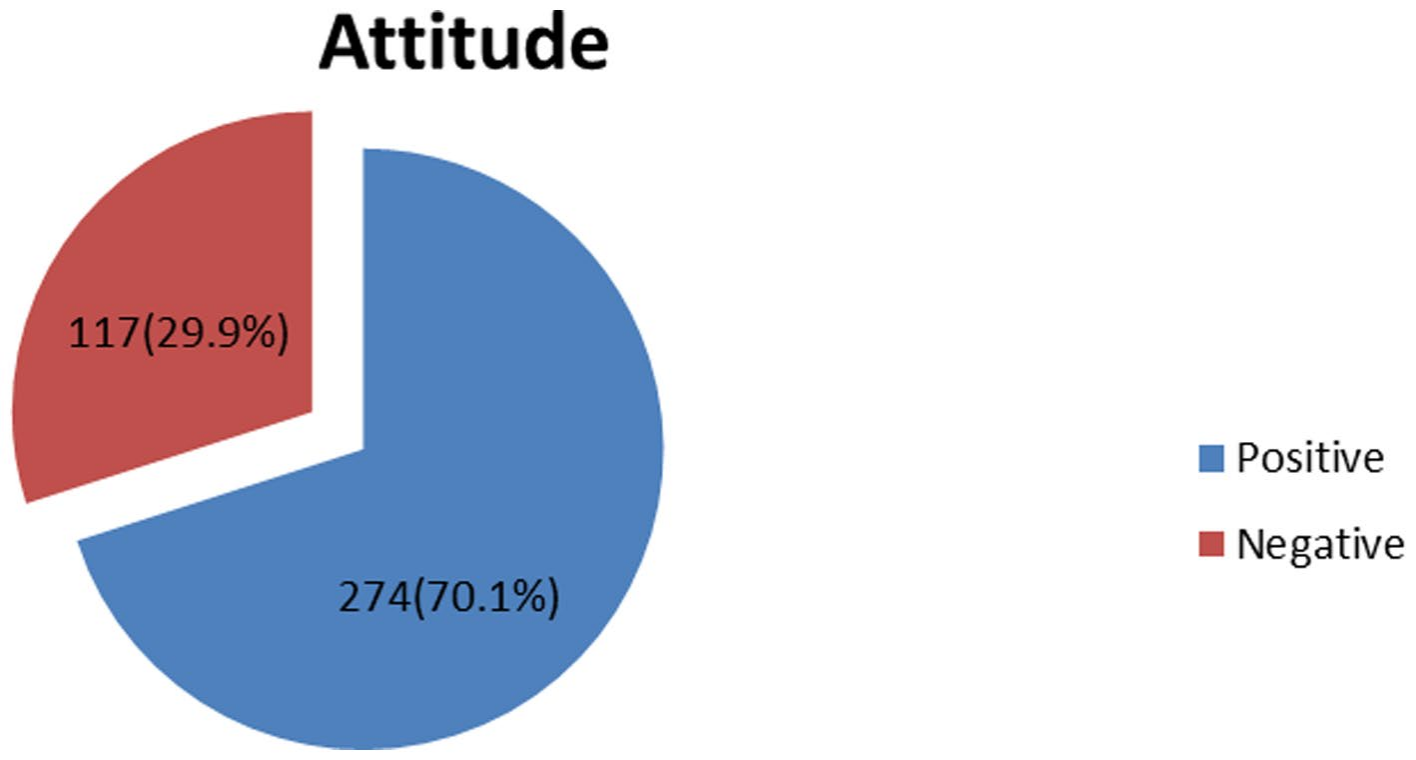
A diagrammatic presentation of magnitude of negative attitude.

A total of 117 (29.9%) respondents have negative attitude toward depression (Figure 3).

Nearly half (46%) of respondents reported an increase in the number of patients presenting with depressive symptoms during the last 5 years.

The majority (88.2%) agreed that a preceding stressors was the cause of the bulk of depressed cases; nevertheless, the majority (78.8%) believed that biochemical abnormality was the cause of more severe sadness. The majority of participants (87.7%) believed that depressed individuals were more likely than other people to have been deprived of basic necessities as children. However, approximately fourth of the respondents (26.3%) felt that patients with depression are discriminated and avoided by the general public.

More than one-third (34.3%) of the respondents agreed that it is difficult to differentiate unhappiness from a clinical depressive disorder that needs treatment. The majority (65%) agreed that using antidepressants to treat depressed people typically results in satisfactory results. Nearly half (48.8%) believed that most depressive disorders improve without medication. Almost a third (61.1%) of respondents agreed with the statement that PHCPs might be a good resource for helping depressed patients. The vast majority of medical professionals (81.3%) agreed that individuals with depression are best off seeing psychiatrists if they need medications or psychotherapy. Most (83.6%) of HCPs felt comfortable dealing with depressed patients; However, approximately 45.8% of respondents concurred that treating depressed patients is “hard going,” tiresome, or challenging. On the other hand, over two-thirds agreed with the claim that caring for depressed people is gratifying (Table 4).

**Table 4:** Depression attitude questionnaires for healthcare professionals working at primary healthcare facilities of Illu Aba Bor zone.

No	Items	Agree *n* (%)	Disagree *n* (%)	Do not know *n* (%)
1	During the last 5 years I have seen an increase in the number of patients presenting with depressive symptoms.	180 (46.0)	59 (15.1)	152 (38.9)
2	Most of the depression cases I see were brought on by recent catastrophe	345 (88.2)	39 (10)	7 (1.8)
3	More severe depression is caused by biochemical abnormalities.	308 (78.8)	75 (19.2)	8 (2.0)
4	Depression can be divided into two categories: one produced by psychological factors and the other by physiological ones.	328 (83.9)	55 (14.1)	8 (2.0)
5	Patients with depression are more likely than other people to have grown up in a deprived environment.	343 (87.7)	48 (12.3)	–
6	People with low stamina use depression as a coping mechanism for their problems.	234 (59.8)	150 (38.4)	7 (1.8)
7	Depression is a natural part of becoming old	123 (31.5)	268 (68.5)	–
8	Are those who suffer from depression avoided and treated differently?	103 (26.3)	281 (71.9)	7 (1.8)
9	Its difficult to tell the difference between sadness and a clinical depression that requires treatment	134 (34.3)	230 (58.8)	27 (6.9)
10	Depression indicates a recognizable reaction that cannot be altered.	92 (23.5)	268 (68.5)	31 (7.9)
11	In normal practice, antidepressants successfully cure depressed individuals in the majority of cases.	254 (65.0)	92 (23.5)	45 (11.5)
12	The majority of depressive disorders get well on their own.	191 (48.8)	157 (40.2)	43 (11.0)
13	The main healthcare provider may be able to help people who are depressed.	239 (61.1)	137 (35.0)	15 (3.8)
14	When depressed people do not improve after receiving treatment from primary healthcare providers, there is not much they can do.	173 (44.2)	187 (47.8)	31 (7.9)
15	It is preferable for depressed people to see psychiatrists rather than primary care providers, if they need antidepressants.	318 (81.3)	54 (13.8)	19 (4.9)
16	Patients with depression should only receive psychotherapy from professionals.	270 (69.1)	101 (25.8)	20 (5.1)
17	Most depressed individuals would benefit more from psychotherapy than medications if it were freely available.	300 (76.7)	83 (21.2)	8 (2.0)
18	Dealing with patients who are depressed comes naturally to me.	327 (83.6)	56 (14.3)	8 (2.0)
19	Working with depressed patients is demanding, tiresome, or challenging.	179 (45.8)	196 (50.1)	16 (4.1)
20	Spending time with sad patients is fulfilling.	281 (71.9)	88 (22.5)	22 (5.6)

### Factors associated with inadequate knowledge toward depression

In bivariate binary logistic regression, variables such as age, educational level, years of experience, working experience in psychiatric units, contact with persons with mental illness, and mental health training were found to have a *p*-value lower than 0.2. These variables fulfilled minimum requirements for further multivariate binary logistic regression.

In multivariate binary logistic regressions, variables such as contact with persons with mental illness and mental health training were found to have statistically significant association with inadequate knowledge at a *p*-value less than 0.05.

The odds of having inadequate knowledge among respondents who have no contact with persons with mental illness was 2.31 times as compared to those who have contact with persons with mental illness (AOR = 2.31, 95% CI; 1.51, 3.20).

The odds of having inadequate knowledge among respondents who have mental health training was 3.32 times as compared to those who have no contact with persons with mental illness (AOR = 3.32, 95% CI; 2.23, 5.98) (Table 5).

**Table 5:** Bivariate and multivariate logistic regression analysis showing association between inadequate knowledge and associated factors among health professionals working at health facilities of Ilu Aba Bor zone, Oromia, Ethiopia, 2021.

Explanatory variables	Knowledge		COR (95%CI)	AOR (95%CI)	*p*-value
	Inadequate	Adequate			
Age					
18–24	12	7	2.47 (1.84, 4.86)	1.88 (0.85, 2.15)	
25–34	89	239	0.54 (0.23, 1.15)	0.77 (0.49, 1.66)	
≥45	18	26	1	1	
Educational level					
Diploma	55	104	1.41 (1.09,3.54)	1.07 (0.47, 2.45)	
BSc degree and above	64	170	1	1	
Experience in years					
<5 years	51	74	2.01 (1.28, 2.97)	1.35 (0.63, 2.17)	
5 years and above	68	198	1	1	
Have contact with person with mental illness					
Yes	59	202	1	1	**0.002**
No	60	70	2.93 (2.22,4.36)	**2.31 (1.51, 3.20)**	
Experience working in psychiatric unit					
Yes	12	53	1	1	
No	107	219	2.16 (1.36, 3.47)	1.18 (0.51, 2.20)	
Training on mental health					
Taken	11	76	1	1	**0.001**
Not taken	108	196	3.81 (2.62, 6.34)	**3.32 (2.23, 5.98)**	

Bold means *p*-value of variables which were significantly associated with inadequate knowledge and unfavourable attitude.

### Factors associated with unfavorable attitude towards depression

In bivariate binary logistic regression, variables such as sex, age, educational level, professional qualification, family history of mental illness, training on mental health, perceived cause of depression, and knowledge levels were found to have a *p*-value less than 0.2. These variables fulfilled minimum requirements for further multivariate binary logistic regression.

In multivariate binary logistic regression, variables such as perceived cause of depression and mental health training were found to have statistically significant association with having negative attitude at *p*-value less than 0.05.

The odds of having an unfavorable attitude among respondents whose traditional or spiritual causes of depression was 2.21 times as compared to those who perceived bio-psychosocial causes for depression (AOR = 2.21, 95% CI; 1.48, 4.63).

The odds of having an unfavorable attitude among respondents who did not have mental health training was 2.87 times as compared to those who have mental health training (AOR = 2.87, 95%CI; 2.16, 4.76) (Table 6).

**Table 6:** Bivariate and multivariate logistic regression analysis showing association between unfavorable attitudes and associated factors among health professionals working at health facilities of Ilu Aba Bor zone, Oromia, Ethiopia, 2021.

Explanatory variables	Attitude		COR (95%CI)	AOR (95%CI)	*p*-value
	Unfavorable	Favorable			
Age					
18–24	11	8	1.81 (1.04, 3.36)	1.23 (0.65, 1.95) 0.36 (0.18, 1.06)	
25–34	87	241	0.48 (0.21, 1.15)	1	
≥45	19	25	1		
Sex					
Male	51	180	1	1	
Female	66	94	2.48 (1.64, 3.35)	1.63 (0.74, 2.08)	
Educational level					
Diploma	61	98	1.98 (1.15, 3.41)	1.11 (0.47, 2.59)	
BSc degree and above	56	178	1	1	
Professional qualification					
Nurse	55	109	2.06 (1.24, 3.53)	1.72 (0.86, 2.33)	
Midwifery	25	46	2.21 (1.57, 3.88)	1.91 (0.94, 2.68)	
Public health	24	66	1.48 (0.85, 1.98)	1.18 (0.57, 1.74)	
Others	13	53	1	1	
Family history of mental illness					
Yes	9	40	1	1	
No	110	232	2.11 (1.36, 3.47)	1.06 (0.51, 2.20)	
Training on mental health					
Taken	12	75	1	1	
Not taken	105	199	3.29 (2.54, 6.11)	2.87 (2.16, 4.76)	**0.001**
Perceived cause of depression					
Traditional or spiritual	27	25	3.87 (2.21, 6.44)	2.21 (1.48, 4.63) 1	
Bio-psychosocial	58	208	1	1.88 (0.87,2.64)	
Both	32	41	2.79 (1.56, 3.67)		**0.002**
Knowledge					
Adequate	67	205	1	1	
Inadequate	50	69	2.22 (1.34, 3.77)	1.43 (0.73, 2.19)	

Bold means *p*-value of variables which were significantly associated with inadequate knowledge and unfavourable attitude.

## Discussion

This study revealed a high level of inadequate knowledge and an unfavorable attitude in the study area in comparison to other studies.

According to this study, 30.4% (95% CI; 25.86, 34.94) of the respondents have inadequate knowledge. This is in line with studies conducted in Ethiopia in which 28.1% of health professionals showed an inadequate knowledge toward depression (36).

The findings in the current study are lower than those of previous studies conducted in Ethiopia (56%) (37), Ethiopia (52.13%) (43), Kenya (62.25%) (12), India (52%) (44), and Lebanon (67.8%) (33). This variation might be due to differences in the tools of assessment employed, variation in socioeconomic status, and educational levels.

Supported by previous studies conducted in Cameroon, the majority of respondents (92.9%) consider depression as a health problem that requires medical treatment (45). The majority (84.1%) view depression as a health issue. The majority of respondents (69.1%) rejected the idea that witchcraft, charms, or evil spirits are to blame for depression. In addition, the majority (88.2%) concurred that recent hardship was the primary cause of the majority of the sad cases they observed. Two-thirds of the participants in a study conducted in Cameroon reported recent bad luck as the main cause of their depression (45).

A significant number of people have blamed non-biopsychosocial factors as the cause of depression. This is an indication that lower care is provided for patients with depression due to minimal depression literacy. This leads to extremely low detection rate of depression by primary care clinician, which poses a serious threat to scaling up mental healthcare in LMICs, which are the countries targeted by the World Health Organization (46).

Those who have no contact with person with mental illness were 2.31 times more likely to have inadequate knowledge as compared to those who have contact with person with mental illness. This is supported by studies in Ethiopia (37) and Saudi Arabia (47). This might be associated with understanding the actual symptoms of depression and the recovery process for those who have it.

Those who do not have mental health training were 3.32 times more likely to have inadequate knowledge when compared to those who have mental health training. This finding is supported by the studies conducted in Ethiopia (31, 34) and Cameroon (45). These studies show that mental health training, including depression, is important to increase the knowledge level of an individual.

According to the current study, the magnitude of the negative attitude toward depression is 29.9% (95% CI; 25.4, 36.8). This is lower than the studies conducted in Ethiopia (44.68%) (43), Zambia (55.6%) (48), Lebanon (67.8%) (33), India (52%) (49), and China (71.3%) (50). This variation might be due to differences in the study population. Some studies included all patients with mental illness, but the current study only included patients with depression. Additionally, differences in tools might have contributed to the variation. The study in India used the Community Attitude towards Mental Illness (CAMI) scale (49), while the current study used questionnaires to measure attitudes toward depression.

Those who perceived traditional or spiritual causes of depression were 2.21 times more likely to have an unfavorable attitude when compared to those who perceived bio-psychosocial causes for depression. It is supported by the study in Ethiopia (31).

According to studies conducted in Ethiopia (31, 35) and Cameroon (45), individuals who have not received mental health training were 3.72 times more likely to have an unfavorable attitude compared to those who have received mental health training.

In the current study, most (83.6%) of the HCPs were comfortable dealing with depressed patients; However, nearly half of the respondents agreed that working with depressed patients is “heavy going,” tedious, or difficult. On the other hand, almost two-thirds agreed with the claim that caring for depressed people is gratifying. According to a survey conducted in China, nearly two-thirds of the respondents concurred that “mental patients frequently destroy property impulsively, and mental patients are burdens to the families and society.” Additionally, primary healthcare providers’ perspectives and competencies in connection to depression have beneficial associations (50).

According to the current study, 290 (74.2%) respondents suggested not to have a close friend with a depressive disorder. Approximately 112 (28.6%) are not willing to live with someone with depressive disorders. This is corroborated by studies conducted in Nigeria, which revealed that more than half of the participants had a close friend who experienced mental health issues. Additionally, one-third of them had neighbors who experienced mental health issues. Furthermore, a quarter of the participants expressed their desire to live with someone who has a mental health issue (51, 52).

Lack of interest to live or work with depressed patients could be due to the wrong perception on its etiology, its treatment outcome, and educational status. This is an implication that those health professionals who have negative attitude to depression will not evaluate patients for depression or cannot provide adequate care for patients, which leads to poor recovery as significant amount of patients who visit primary healthcare setting have untreated depression (53).

The current study has limitations: first, it does not show temporal relationship as it is a cross-sectional study. Second, variables used in the study were limited in number, and finally, the practice domain has not been reported.

## Conclusion

According to this study, the magnitude of inadequate knowledge and negative attitudes toward the diagnosis and treatment of depression are quite prevalent. Different factors were involved in the presentation of the current finding; therefore, it is important to take caution and continue toward improving awareness, changing negative beliefs, and enhancing the detection and management of depression in primary healthcare settings. In addition, it is necessary to facilitate different awareness creation strategies and prepare training for health professionals so as to foster holistic health service delivery.

## Data availability statement

The raw data supporting the conclusions of this article will be made available by the authors, without undue reservation.

## Ethics statement

The studies involving human participants were reviewed and approved by Mattu University College of health sciences ethical review Committee. The participants provided their written informed consent to participate in this study.

## Author contributions

YA took part in the development of the proposal, data collection, data entry, analysis, and manuscript preparation. MG took part in the development of the proposal, data entry, analysis and interpretation, and report writing. LB took part in the development of the proposal, data collection, data entry, analysis, and interpretation. KB took part in the development of the proposal, data entry, data interpretation, and report writing appropriately. HT took part in developing the proposal, analyzing and interpreting the data, and writing the manuscript. All authors contributed to the article and approved the submitted version.

## Glossary

CAMI: community attitude toward mental illness
EBR: Ethiopian Birr
EMOH: Ethiopian ministry of health
HCPs: healthcare providers
HCWs: healthcare workers
HEPs: health extension professionals
HWs: health workers
KAP: knowledge, attitude, and practice
LMICs: low and middle income countries
MAKS: mental health knowledge schedule
MICA: mental illness clinicians’ attitude scale
MhGAP: mental health gap action programme
MHPs: mental health professionals
PHC: primary health care
SMD: severe mental disorders
SPSS: statistical package for social sciences
UK: United Kingdom
WHO: world health organization
